# Evaluation of Circulating Platelet Extracellular Vesicles and Hypertension Mediated Organ Damage

**DOI:** 10.3390/ijms232315150

**Published:** 2022-12-02

**Authors:** Leslie Marisol Lugo-Gavidia, Dylan Burger, Janis M. Nolde, Vance B. Matthews, Markus P. Schlaich

**Affiliations:** 1 Dobney Hypertension Centre, Medical School—Royal Perth Hospital Unit, Royal Perth Hospital Medical Research Foundation, The University of Western Australia, Perth, WA 6000, Australia; 2 Kidney Research Centre, The Ottawa Hospital Research Institute, Department of Cellular and Molecular Medicine, University of Ottawa, Ottawa, ON K1N 6N5, Canada; 3 Department of Internal Medicine, Royal Perth Hospital, Perth, WA 6000, Australia; 4 Departments of Cardiology and Nephrology, Royal Perth Hospital, Perth, WA 6000, Australia

**Keywords:** platelet extracellular vesicles, endothelial function, organ damage, hypertension

## Abstract

Elevated circulating platelet-derived extracellular vesicles (pEVs) have been associated with arterial hypertension. The role of hypertension-mediated organ damage (HMOD) to induce EV release is still unknown. We studied the micro- and macro-vascular changes (retinal vascular density and pulse wave velocity), endothelial function (flow-mediated vasodilation of brachial artery and finger plethysmography), and assessed the psychosocial status (anxiety and depression) in hypertensive patients to determine their relationship with EV release. Pulse wave velocity showed a significant positive correlation with pEVs (r = 0.33; *p* = 0.01). Systolic blood pressure (SBP) negatively correlated with retinal vascularity. The superficial retinal vascular plexus density in the whole image showed a significant negative correlation with 24 h SBP (r = −0.38, *p* < 0.01), day-SBP (r = −0.35, *p* = 0.01), and night-SBP (r = −0.27, *p* = 0.04). pEVs did not show significant associations with microvascular damage (retinal vascular density), endothelial function (flow-mediated vasodilation of brachial artery and finger plethysmography), or psychosocial status (anxiety and depression). Our results indicate that the pEV levels were associated with macrovascular damage measured by PWV, whereas no significant association between pEVs and microvascular damage, endothelial function, or emotional status could be detected. The potential utility of pEV in clinical practice in the context of HMOD may be limited to macrovascular changes.

## 1. Introduction

Hypertension (HTN) is one of the most prevalent cardiovascular risk factors. The increased mechanical stress induces structural and/or functional changes in major organs, leading to hypertension mediated organ damage (HMOD) [1]. Although costs and technical equipment might limit its access, an appropriate evaluation of HMOD is fundamental as it has been associated with adverse prognosis and can help to reclassify patients that otherwise would be considered low risk. 

In the past decades, extracellular vesicles (EV) have emerged as a potential early biomarker in cardiovascular disease. EVs are small cell vesicles derived from the cell membrane of different cells in response to stress, injury, or cell activation. EV could have an important role on HTN as they have been associated with the underlying mechanisms causing HMOD such as vascular integrity, endothelial function, inflammation, and thrombosis [2]. 

The constant high-pressure flow in HTN has been shown to affect the microcirculation, endothelial function, and promote a thrombogenic state. The latter may be particularly important given that thrombotic events represent one of the most detrimental complications of hypertension [1,3,4]. As a result, extensive investigations have explored the numerous factors affecting platelet reactivity. Whilst diabetes mellitus, high-cholesterol, smoking status, and other factors [5] have been related to thrombotic risk, other conditions such as psychological stress have been somewhat neglected in this area, despite evidence of its association through plasminogen activator inhibitor (PAI)-1 and chronic inflammation [6]. Most cardiovascular risk calculators are based on classical-risk factors, however, recently, the use of additional information such as imaging, functional tests, biomarkers, and psychosocial evaluation have been proposed as tools to improve the assessment of cumulative organ damage and more accurately capture the long-term progression and impact of the disease. The evaluation of platelet-derived extracellular vesicles (pEVs) represents an interesting option in this framework in light of their strong pro-coagulant capacity [2,7,8,9]. Additionally, pEVs have the ability to facilitate atherogenesis, and their surface markers and cargo also modulate multiple effects implicated in endothelial function and inflammation through the delivery of bioactive molecules and/or cell-to-cell interaction [10]. 

**Figure 1:**
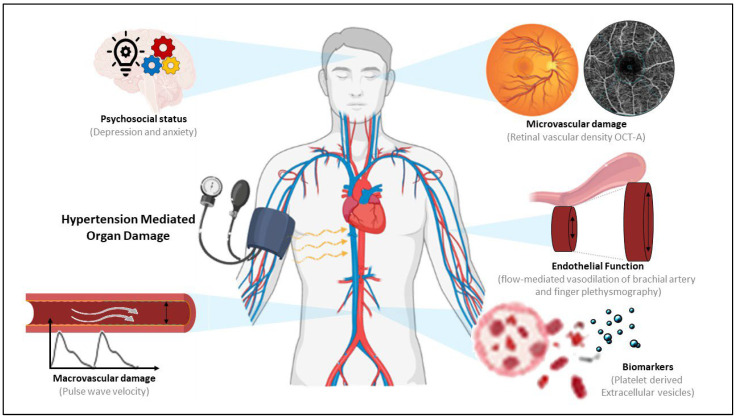
**Schematic representation of the study techniques to evaluate hypertension organ damage (HMOD).** Pulse wave velocity was used to assess arterial stiffness as a marker of macrovascular damage. Vascular density in different layers of the retina using optical coherence tomography–angiography (OCT–A) was used to evaluate microvascular damage. Flow-mediated vasodilation of the brachial artery and finger plethysmography were used to determine endothelial function. Indices of anxiety and depression were used to determine the psychosocial status. Platelet derived extracellular vesicles were evaluated as a possible biomarker of vascular damage.

While the association of pEV and classical clinical characteristics has been investigated previously, the evaluation of pEV with subclinical damage can provide a better insight into their role as an early biomarker. In order to investigate the potential use of pEV in HTN, we used state-of-the-art techniques in hypertensive patients to evaluate early HMOD and psychosocial status. We evaluated macrovascular damage by pulse wave velocity (PWV), the gold standard to evaluate arterial stiffness. Furthermore, we assessed microvascular changes evaluated by vascular density in different layers of the retina using optical coherence tomography–angiography (OCT–A), a modern technology to complement retinal assessment and reliably detect early microvascular damage. Additionally, we used non-invasive techniques to measure endothelial function (flow-mediated vasodilation of brachial artery (FMD) and finger plethysmography (EndoPAT)) (Figure 1). These state-of-the-art techniques are a growing field for cardiovascular assessment and can provide more detailed information about early subclinical cardiovascular damage that otherwise might go undetected. 

## 2. Results

### 2.1. Platelet Derived Extracellular Vesicles and Macro/Microvascular Organ Damage 

The baseline demographics of the study population are summarized in Table 1. The study population had a mean age of 53.9 ± 15.2 years and included 64.5% males. Hypertension was present in 83.9% and diabetes was diagnosed in 30.6% of the study population. The mean office BP across the overall population was 128 ±14.8/77.3 ± 12.1 mmHg.

The mean level of the platelet-derived EVs were higher in patients with hypertension compared to normotensives (10.9 ± 6.70 vs. 6.3 ± 4.43 EV/µL; *p* = 0.03). Normotensive participants showed less pronounced macrovascular damage with significative lower PWV and AIx than hypertensive patients. PWV showed a significant positive correlation with pEVs (r = 0.33; *p* = 0.01). PWV showed a significant correlation with systolic blood pressure (SBP) measured in the office (r = −0.50, *p* < 0.001), 24 h (r = −0.46, *p* < 0.001), day-time (r = −0.50, *p* < 0.001), and night (r = −0.60, *p* < 0.001).

**Figure 2:**
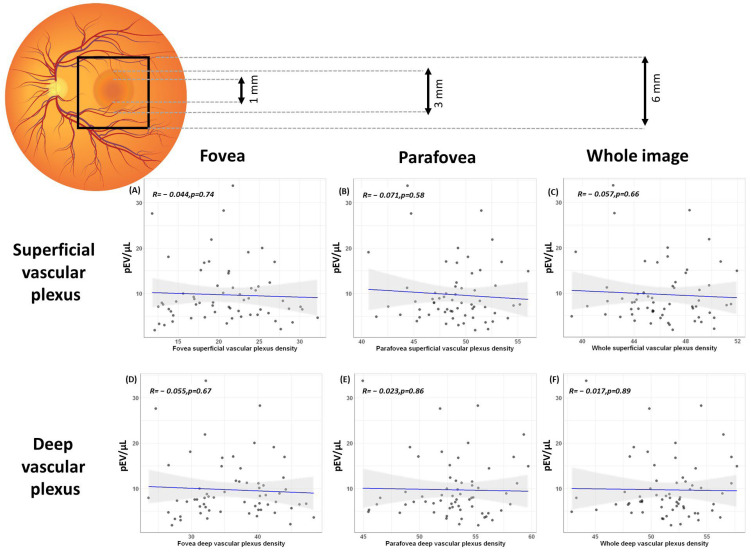
**Platelet derived extracellular vesicles and retinal vascular density. Top panel.** Schematic representation of the retina and the definitions for the zones employed in this study. The foveal density refers to the 1 mm diameter circle around the fovea, parafoveal to a ring from 1 mm to 3 mm around the fovea and whole image to everything within the 6 × 6 mm square centred on the fovea. **Centre panel.** Scatterplot of extracellular vesicles and superficial vascular plexus density at (**A**) fovea zone (*p* = 0.74), (**B**) parafovea zone (*p* = 0.58), and (**C**) whole image (*p* = 0.66). **Bottom panel.** Scatterplot of extracellular vesicles and deep vascular plexus density at (**D**) fovea zone (*p* = 0.67), (**E**) parafovea zone (*p* = 0.86), and (**F**) whole image (*p* = 0.89). Blue line represents the regression line of best fit. The grey bands around the line represents 95% confidence intervals.

In contrast, microvascular damage evaluated by retinal vascular density did not show any significant differences between normotensive and hypertensive patients (Table 2). Furthermore, pEVs did not show any significant correlation with the superficial vascular plexus density (fovea zone (*p* = 0.74), parafovea zone (*p* = 0.58), and whole image (*p* = 0.66)) or the deep vascular plexus density (fovea zone (*p* = 0.67), parafovea zone (*p* = 0.86), and whole image (*p* = 0.89) (Figure 2).

Interestingly, the superficial vascular plexus density in the whole image showed a significant negative correlation with 24 h SBP (r = −0.38, *p* < 0.01), day-SBP (r = −0.35, *p* = 0.01), and night-SBP (r = −0.27, *p* = 0.04). Office BP did not show a significant association. The parafoveal superficial vascular plexus density also showed a significant negative association with 24 h SBP (r = −0.37, *p* = 0.01) and day-SBP (r = −0.34, *p* = 0.01), but not with night-SBP or office BP. No other significant associations were found in the rest of the retinal areas. 

### 2.2. Endothelial Function and Platelet Derived Extracellular Vesicles

**Figure 3:**
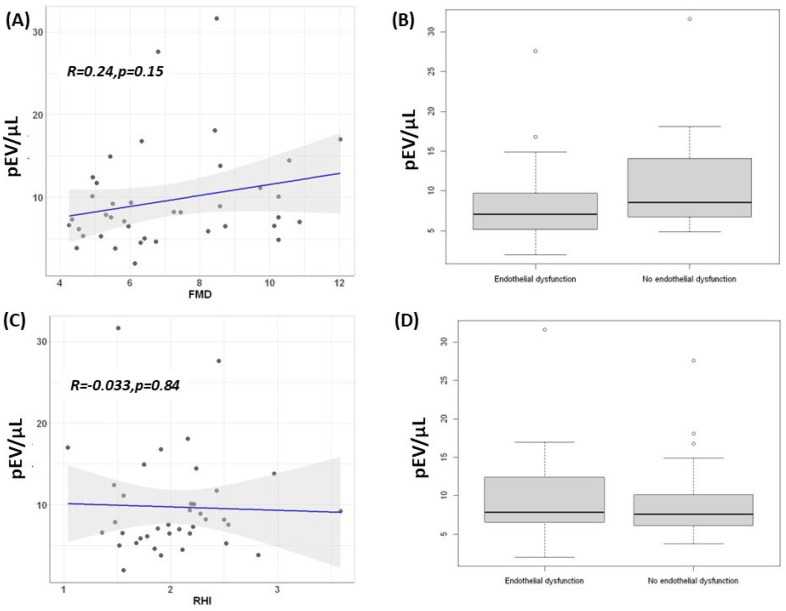
**Platelet derived extracellular vesicles and endothelial function.** (**A**) Scatterplot of extracellular vesicles and flow mediated dilatation (*p* = 0.15). (**B**) Boxplot of extracellular vesicles and patients with and without endothelial dysfunction according to the FMD measurements (*p* = 0.19). (**C**) Scatterplot of the extracellular vesicles and reactive hyperaemia index (*p* = 0.84). (**D**) Boxplot of the extracellular vesicles and patients with and without endothelial dysfunction according to the reactive hyperaemia index measurements (*p* = 0.57). Blue line represents the regression line of best fit. The grey bands around the line represents 95% confidence intervals.

The endothelial function measured by FMD had a mean of 7.08 ± 2.18% (min 4.26%–max 12.03%) and did not show any correlation with EV (R = 0.24, *p* = 0.15) (Figure 3). The patients were classified according to the presence of endothelial dysfunction (FMD < 7.1%) [11]. Twenty-three patients (58%) had endothelial dysfunction. No significant differences were found in the pEV or blood pressure levels (office or ambulatory) (Table 3). 

Similarly, RHI evaluated by finger plethysmography was not correlated with the levels of pEV (r = −0.03, *p* = 0.84). Patients were evaluated for endothelial dysfunction according to the levels of RHI. Interestingly, only nine patients fulfilled the criteria for endothelial dysfunction (RHI < 1.67) [12] with finger plethysmography. No significant differences were found when comparing both groups. (Figure 3 and Table 3).

### 2.3. Emotional Status and Platelet Derived Extracellular Vesicles

Finally, we evaluated the emotional status in the hypertensive spectrum. Night-SBP showed a significant negative correlation with anxiety score (trait) (r = −0.33; *p* = 0.02). In contrast, the anxiety score did not show any significant correlation with systolic AOBP (r = −0.17; *p* = 0.22), 24 h-BP (r = −0.22; *p* = 0.12), or day-BP (r = −0.12; *p* = 0.41). The anxiety score (state) did not show any significant correlation with systolic AOBP, 24 h-BP, day-BP, or night-BP. No significant correlation was found between the EV and anxiety scores.

**Figure 4:**
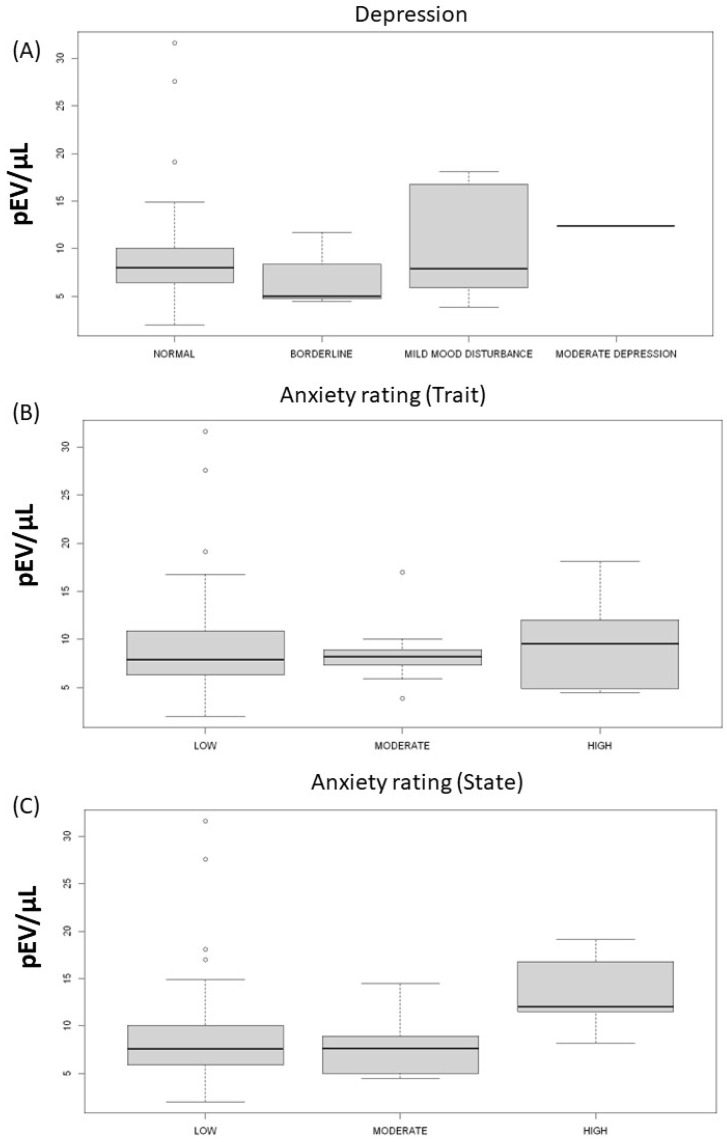
**Platelet derived extracellular vesicles and emotional evaluation.** (**A**) Boxplot of depression categories and extracellular vesicles (*p* = 0.83). (**B**) Boxplot of anxiety rating (trait) categories and extracellular vesicles (*p* = 0.88). (**C**) Boxplot of anxiety rating (state) categories and extracellular vesicles (*p* = 0.21).

The patients were classified according to their anxiety scores in low, moderate, and high anxiety. Overall, patients with high anxiety levels (trait) showed a trend to lower values of BP, this difference was significant in systolic blood pressure levels at night-time. In contrast, patients presenting high anxiety levels (state) did not express this pattern, furthermore, diastolic blood pressures during night-time showed significantly higher levels in patients with high anxiety. Patients with high anxiety levels (state) showed a trend to higher levels of pEV. Stress markers did not show any statistical associations (Table 4 and Figure 4).

The depression score did not show any significant correlation with blood pressure levels, pEV, or stress markers. There was no significant difference between the depression categories in any parameter.

## 3. Discussion

The major findings of our study are: (a) patients with hypertension showed higher levels of pEV than normotensive participants; (b) PWV showed a significant correlation with pEVs and SBP; (c) parafoveal and whole superficial vascular density were inversely associated 24 h SBP and day SBP. Nocturnal SBP was also inversely associated with whole vascular density. (d) pEVs did not show significant associations with microvascular damage (retinal vascular density), endothelial function (flow-mediated vasodilation of brachial artery and finger plethysmography), and psychosocial status (anxiety and depression).

Using gold standard BP evaluation (office and ambulatory BP). we have previously demonstrated a positive correlation between the pEV levels and BP and PWV [13,14,15]. Other studies have previously investigated EVs in the context of vascular damage and blood pressure, with a special focus on endothelial-derived EVs. In this analysis, we used different state-of-the-art techniques to evaluate different aspects of HMOD to represent an integrated overview of the underlying mechanisms of vascular damage and its relationship with platelet-derived EV. The latter may be particularly important given their thrombogenic properties and the fact that thrombotic events represents one of the most detrimental complications of hypertension [2,3,4,10].

Arterial stiffness has been widely recognized as a surrogate for cardiovascular disease, and current guidelines recommend the assessment of arterial stiffness to evaluate HMOD and underlying arteriosclerosis, especially in patients who appear to be asymptomatic. A PWV >10 m/s is considered to represent significant alterations in the arterial elastic properties and determine subclinical HMOD [1,16,17,18]. Some studies have suggested an additive value of PWV for cardiovascular risk estimation, however, to date it is not routinely performed in clinical practice [19,20]. In our population, we demonstrated increased values of PWV and pEV in patients presenting HTN compared to the normotensive participants, with a positive correlation between the PWV and pEV levels. These results suggest an association of pEVs with macrovascular damage. This is consistent with the findings of Wang et al., who reported that values of brachial-ankle pulse wave velocity were robustly associated with circulating levels of endothelial-derived EV [21].

In contrast, when we evaluated the microvascular damage by retinal vascular density, we did not find a significant association with the levels of pEVs. While no significant differences were found in the vascular density between the normotensive and hypertensive groups, a significant correlation was found between the superficial vascular density and ambulatory systolic BP measurements in the whole image and in the parafoveal portion, but not in the deep vascular plexus. Retinal evaluation is recommended in hypertension for its prognostic significance and should be performed routinely in grades 2 or 3 hypertension [1]. New imaging techniques such as OCT–A represent a promising tool to assess additional parameters in the individual layers of the retina [22]. Several studies have related OCT–A parameters in hypertensive patients [23,24,25,26]. In our analysis, the negative correlation of ambulatory BP measurements with the superficial plexus but not with office BP reflects the superior association of ambulatory BP measurements with early HMOD. The lack of association between BP and the foveal and deep vascular plexus density suggests that the changes in these areas might be related to a much more severe progression of the disease. While our population included blood pressure levels across the entire blood pressure spectrum (normotension, mild-, moderate-, and severe-BP levels), the number of patients with severe hypertension was limited, which also resulted in less clinical symptomatic organ damage.

Endothelial dysfunction is considered as an early feature of atherosclerosis. There is now overwhelming evidence associating endothelial function with cardiovascular risk factors, which has motivated its use as a therapeutic target for cardiovascular disease prevention [27,28,29,30,31]. In the context of hypertension, patients present impaired endothelial-dependent vasodilation. The disturbed blood flow and altered haemodynamics are the main mechanisms responsible for the impairment of the vascular endothelium in HTN [27,28,30]. Several studies have shown endothelial dysfunction in patients with hypertension compared to healthy controls [32] and their relationship with the progression of the disease [33,34]. However, a larger study in a multi-ethnic cohort showed FMD not to be a significant independent predictor of hypertension in the adjusted model [35], which highlights the role of different risk factors in endothelial dysfunction. Given that EVs can be derived from endothelial cells, there is a great interest in understanding their implication in endothelial function. Sansone et al. reported elevated concentrations of endothelial EV in patients with hypertension and its relationship with FMD. Furthermore, they observed an inverse correlation between the change in FMD and endothelial EVs after BP normalization during hypertensive emergencies [36]. Similar results were found by Horn et al., showing a correlation between the increase in FMD and the decrease in endothelial-derived EV levels after transcatheter aortic valve implantation [37]. In our analysis, we studied platelet-derived EV, as one of the key characteristics of endothelial dysfunction is to confer a prothrombotic phenotype. There was no significant correlation between the endothelial function (FMD or RHI) and platelet-derived EVs in our population. Additionally, no differences were observed when the patients were categorized by the presence or absence of endothelial dysfunction. The differences in our results could be explained by the type of EV analysed and the characteristics of the patients, as the patients included in the Sansone and Horn studies presented more severe conditions (e.g., hypertensive emergencies, severe aortic stenosis), which directly confer more severe endothelial dysfunction (FMD ~0–8%) [36,37]. Our population had a wider range of FMD values (min 4.26%–max 12.03%) and less pronounced endothelial damage. Some other technical issues need to be taken into account when interpreting the results: (a) FMD has an inter and intra-observer variability; (b) there are different protocols that complicate the comparisons with other centres [27]; (c) we also used the corrected %FMD by allometric scaling to obtain a more accurate FMD ratio to account for the confounding influence of allometric scaling between individuals. No statistical associations with EV and RHI measured by finger plethysmography were found, confirming the results obtained with FMD; however, there was a reduction in the number of patients presenting endothelial dysfunction defined by RHI cutoff values compared to FMD, so it is important to remember that finger plethysmography is dependent on different non-endothelial factors [27].

Finally, we investigated the association between emotional status and pEV release. Anxiety and depression have been commonly described in patients with cardiovascular disease and may significantly influence their overall health and quality of life. The link between emotional status and cardiovascular disease is complex and is not fully understood. The emotional status may be a normal response to the patient’s current situation; however, excessive and long-standing emotional stress is considered detrimental [6,38]. Chronic dysregulation of autonomic function, inflammation, endothelial dysfunction, and platelet activation are some of the suggested mechanisms linking emotional status with cardiovascular disease. Depression has been shown to present elevated plasminogen activator inhibitor (PAI)-1 [39], a major contributor to thrombosis. Additionally, PAI-1 is also involved with stress-related neural remodelling and the hypothalamic–pituitary–adrenal axis regulation [6,40,41]. Additionally, patients presenting anxiety have shown higher levels of PAI-1 and composite haemostatic score in previous studies [41]. These results seem to indicate an influence of the emotional status with the thrombotic system. In our study, we investigated the relationship between anxiety and depression with platelet-derived EVs, which have been suggested as an early thrombotic marker due to their procoagulant capacities. pEV did not show any association with the emotional status, although patients with high anxiety (state) showed a trend to higher levels of EV. Surprisingly, anxiety (trait) was inversely correlated with values of nocturnal blood pressure, and patients with high anxiety showed lower levels of SBP at night-time. While it would be expected that patients with higher levels of anxiety present higher levels of blood pressure due to higher sympathetic activity, the results in our analysis can be explained by the fact that patients with anxiety traits most commonly displayed a deeper concern for their health, and therefore have stricter control of their blood pressure. This is a hypothesis that needs to be proven in larger studies.

Our study had some limitations. First, given the sample size and the cross-sectional nature of this analysis, a causal relationship cannot necessarily be inferred and can only be interpreted as hypothesis-generating. Second, as we used state-of-the-art techniques (OCT–A, Endopat, FMD) as a surrogate of HMOD, studies including hard outcomes are required to fully understand their prognostic value. We acknowledge the inherent limitations of this investigation due to its observational nature.

In conclusion, our results indicate that the pEVs levels were associated with macrovascular damage measured by PWV. We did not find any significant association between pEVs and microvascular damage, endothelial function, or emotional status. While recent investigations indicate that pEVs have a role in the underlying mechanisms of cardiovascular disease, their use in clinical practice is still limited and may be restricted to specific subpopulations.

## 4. Materials and Methods

### 4.1. Subject Population and Study Design 

Patients between 18 and 85 years old were recruited from the outpatient hypertension clinic of the Royal Perth Hospital Medical Research Foundation. The study complied with the Declaration of Helsinki and received approval from the University of Western Australia research ethics committee. All participants provided written consent for the study. Clinical baseline data were collected from the patients including medical history, medication history, serum pathology, and blood pressure (BP) evaluation by office and ambulatory blood pressure measurements (24 h BP, day BP, and night BP). All patients were referred to undergo extended testing including macro- and microvascular damage, endothelial function, and emotional evaluation, however, if the test could not be completed (e.g., medical contraindication for prolonged ischemia, patient refusal to mental health evaluation, etc.) or the quality of the data was insufficient for a correct interpretation, the patient was excluded from the specific test analysis.

In the first analysis, we investigated pEV levels along with microvascular and macrovascular damage in the blood pressure spectrum (normotension, mild, moderate, and severe-BP levels). We included 62 subjects, 10 normotensive and 52 hypertensive subjects. HTN was defined by the elevated office and/or ambulatory blood pressure or history of hypertension with ongoing antihypertensive medication. Retinal vascular density was evaluated in the superficial and deep vascular plexus by optical coherence tomography–angiography (OCT–A). Urine albumin–creatinine ratio was measured as a surrogate of renal microvascular damage. Arterial stiffness was used as a marker of macrovascular damage and was determined by PWV. In view of the absence of established reference values for vascular density and EV, the normotensive group served as a reference group to put vascular density and EV values from our cohort into perspective and enable clinical interpretation.

In the second analysis, we analysed endothelial function in 39 hypertensive patients. Participants underwent endothelial function testing by flow-mediated dilatation (FMD) and finger plethysmography (EndoPAT). The patients were subsequently divided into two groups according to the presence of endothelial dysfunction using the previously reported cutoff values for each test [11,12].

In the third series, we assessed the pEV levels in relation to the emotional status (anxiety and depression) in 49 hypertensive patients. Additionally, we assessed cortisol and high sensitivity C-reactive protein as biological stress-related markers. 

Platelet EV (CD41^+^/Annexin V^+^) were analysed by flow cytometry according to the expression of surface antigens. Patients who had heart failure NYHA class III–IV, chronic kidney disease (eGFR of <30 mL/min/1.73 m^2^), or active autoimmune disease requiring treatment with corticosteroids or other immunosuppressive agents were not eligible to be included in any of the studies.

### 4.2. Blood Pressure Evaluation

Office blood pressure from the brachial artery was measured according to international guidelines. Automated blood pressure was measured after 5-min of resting in the sitting position three times with one minute rest periods between measurements (HEM 907 Automatic Blood Pressure Monitor^®^; Omron Healthcare Co., Kyoto, Japan). Unattended automated office blood pressure (AOBP) was defined as the average of the three measurements.

Ambulatory blood pressure monitoring (ABPM) was performed throughout 24 h with clinically validated devices (Spacelabs healthcare, Issaquah, WA, USA; Mobil-O-Graph IEM GmbH, Stolberg, Germany; OSCAR SunTech, Morrisville, NC, USA). The device was set to measure BP every 15 min during day-time (6:00 h–22:00 h) and every 30 min during night-time (22:00 to 6:00 h). The 24 h-BP, day-BP, and night-BP were reported as the average of successful readings recorded during the period. Participants were instructed to follow their usual daily activities but remain still during the measurement. Daily activities were documented in a printed diary including bedtimes (adjustment of awake and asleep periods was made if required) and medication intake. Only patients with successful readings were included in the 24 h-BP, day-BP, and night-BP analysis (minimum of 70% successful readings including 20 day-time and seven night-time) [42]. 

### 4.3. Vascular Density

Retinal imaging was performed using the Optovue AngioVue^®^ spectral-domain optical coherence tomography–angiography (OCT–A) device (Optovue, Inc., Fremont, CA, USA). Vascular density (VD) was estimated non-invasively with OCT–A technology via time-dependent signals from the laser reflection of flowing erythrocytes. VD was defined as the percentage of sample area (whole scan size was 6 × 6 mm) occupied by vessel lumens following the binary reconstruction of images. An automated analysis tool of AngioVue software (version 2018.1.0.43) Fremont, CA, USA was used to calculate the VD in the superficial and deep vascular plexus. The fovea centre was automatically determined, and VD was determined in the whole image (6 × 6 mm), inner and outer rings representing the foveal (1 mm diameter around the fovea) and parafoveal (ring from 1–3 mm around the fovea), respectively.

OCT–A scans were reviewed by a trained operator to ensure sufficient image quality. The average of both eyes was used to define the individual VD for each section. If the scans of any of the eyes could not be performed (e.g., clinical contraindication) or the quality was too low for assessment, only the scans of the remaining eye were used (if both eyes did not reach sufficient quality, the patient was excluded from analysis).

### 4.4. Arterial Stiffness Evaluation

Arterial stiffness was assessed by non-invasive pulse wave analysis (PWA) and pulse wave velocity (PWV) performed with the SphygmoCor XCEL system (AtCor Medical Pty Ltd., Australia) in accordance with the manufacturer’s recommendations. PWA was performed after a 5 min rest period in the supine position, and an automatic 10 s PWA reading was used for data acquisition. Simultaneous measurements through applanation tonometry over the carotid and femoral artery provided the pulse transit time. The time that elapsed between the carotid and femoral artery sites was used to calculate the pulse wave velocity. The capturing time for the PWV assessment was set to 10 s with a PWV distance and subtraction method. PWV assessments were performed twice, and their average was used for further analysis. PWV was expressed as the distance/transit time (m/s). Several hemodynamic parameters were documented including central mean arterial pressure (cMAP), aortic augmentation pressure (AP), and augmentation index (AIx). AIx was normalized for a heartbeat of 75 beats per minute to enable comparations and was expressed as a percentage.

### 4.5. Endothelial Function

FMD was performed on patients who fasted for 6 h, in a temperature-controlled room after 15 min of supine rest. FMD was assessed by high-resolution B-mode ultrasound, images were recorded every second and analysed with an automated analysis system (Brachial Analyzer, Vascular Research Tools, Medical Imaging Applications LLC, Coralville IA). The patient’s arm was extended and positioned at an angle of 80° from the torso. A forearm blood-pressure cuff was placed distal to the cubital fossa. Baseline diameter was measured before cuff inflation for a period of 1 min. The forearm cuff was inflated (>200 mmHg) for 5 min. Diameter and flow recordings were resumed 30 s before cuff deflation and continued for 3 min after. All images were stored as AVI files for offline analysis. The brachial maximal diameter was determined as the 5 s average peak diameter observed during the plateau phase after cuff deflation. FMD was defined as the difference in arterial diameter from the baseline diameter (*D_baseline_*) to a postischaemic maximal diameter (*D_max_*) expressed as a percentage of *D_baseline_*:

$$\%FMD=\frac{\left(Dmax-Dbaseline\right)}{Dbaseline}\times 100$$

We used the corrected %*FMD* by allometric scaling to obtain a more accurate *FMD* ratio as it accounts for the confounding influence of allometric scaling between individuals [43].

Endothelial function was also assessed by finger plethysmography (EndoPAT). Digital pulse amplitude was measured with a pulse amplitude tonometry (PAT) device placed on the tip of each index finger (Itamar Medical). PAT was assessed in response to reactive hyperaemia. Measurements were obtained for 5 min at baseline followed by 5 min of occlusion of one arm, with a blood pressure cuff inflated on the upper arm to supra-systolic pressure (60 mmHg above systolic pressure or 200 mmHg) and then released to induce reactive flow-mediated hyperaemia, which was measured for 5 min. The reactive hyperaemia index (RHI) was calculated as the ratio of the average amplitude of the PAT signal over a minute time interval, one minute post deflation divided by the average amplitude of the PAT signal taken for 3.5 min during the baseline, corresponding to the automatic algorithm provided by Itamar Medical. 

### 4.6. Emotional Status Assessment

The emotional status was determined using the Beck Depression Inventory (BDI-II) to assess symptoms of depression. Patients were subsequently classified according to their score as normal (1–10), mild mood disturbance (11–16), borderline clinical depression (17–20), moderate depression (21–30), severe depression (31–40), and extreme depression (>40).

The Spielberger’s State and Trait Anxiety inventories were used to determine the level of anxiety. Patients were categorized as having no or low anxiety (20–37), moderate anxiety (38–44), and high anxiety (45–80) [44].

### 4.7. Platelet EV Characterization

Platelet EV subpopulations were evaluated by flow cytometry according to the expression of platelet markers (CD41) as described previously by our group. [45] Briefly, venous blood was collected after 10–12 h of fasting into 3.8% sodium citrate tubes. The first 3 mL of blood was discarded to avoid platelet activation. Platelet-free plasma (PFP) was obtained by successive centrifugation at 800× *g* for 10 min and double centrifugation at 2500× *g* for 15 min at room temperature (RT). PFP was immediately frozen and stored at −80 °C until processing for isolation and quantification. All samples were processed identically and within 1 h after extraction. Samples that failed to accurately measure pEVs (e.g., insufficient volume or hemolysis) were excluded from the analysis.

To isolate large pEVs, PFP frozen aliquots were thawed at RT and centrifuged at 12,000× *g* for 2 min to remove fibrin clots/aggregates. The supernatant (400 μL) was collected for subsequent high-speed centrifugation at 20,000× *g* for 20 min. The supernatant was discarded, and the remaining pEV-enriched pellet was re-suspended in 300 μL ultrafiltered PBS. Re-suspended pEVs were incubated for 60 min with fluorochrome-labelled antibodies (CD41-PE-Cy7). The mix was subsequently incubated with Annexin V-FITC at 5% for 10 min and diluted with ultra-filtered annexin binding buffer (10 mM HEPES, pH 7.4, 140 mM NaCl, 2.5 mM CaCl2) before being immediately analysed on an Attune^TM^ NxT Acoustic Focusing Cytometer. Equivalent concentrations of the respective isotype controls were used to determine the degree of non-specific binding. The acquisition was performed using the lower flow rate (12.5 μL/ min). Forward scatter (FSC), side scatters (SSC), and fluorescence data were obtained with the settings in the logarithmic scale. The concentration of pEVs was determined by volumetric cell count in 50 µL of the sample within the gate limits established by ApogeeMix (Apogee Flow Systems). The lower detection threshold was set using the 80 nm fluorescent/180 nm silica beads signal. pEVs within the established gate limits were identified and quantified based on their binding to Annexin V and reactivity to CD41-PECy7 to define platelet-derived extracellular vesicles (Supplementary Materials, Figure S1).

### 4.8. Statistical Analysis

For the baseline characteristics, continuous variables were expressed as the mean ± SD and categorical variables as the frequencies and percentages. Qualitative variables were compared with the chi-square test or Fisher’s exact test if the application conditions were not fulfilled. Differences in quantitative variables between groups were made with an unpaired *t*-test or one-way ANOVA method accordingly. Post hoc Tukey test was performed to evaluate all between-group comparations. The Pearson correlation coefficient was used for correlation analyses for continuous variables. Normality of the data was assessed by the Shapiro–Wilk test. Non-parametric tests were applied when required (e.g., pEV, emotional status). A *p*-value < 0.05 was considered statistically significant for all comparisons. Statistical analysis was performed using R 4.0.3 software. 

## Figures and Tables

**Table 1:** Baseline characteristics of the analysed patient cohort.

Study Population *n* = 62	
Male	40 (64.5%)
Age	53.9 (15.2)
BMI (Kg/m^2^)	29.3 (5.08)
Diabetes	19 (30.6%)
Hypertension	52 (83.9%)
Dyslipidaemia	36 (58.1%)
White cell count (10*9/L)	6.11 (1.56)
Red cell count (10*9/L)	4.81 (0.440)
Haematocrit (L/L)	0.43 (0.03)
Haemoglobin (g/L)	143 (11.1)
Platelet count (10*9/L)	248 (56.7)
Glucose (mmol/L)	5.95 (1.47)
HbA1c (%)	6.15 (1.16)
Total cholesterol (mmol/L)	4.90 (1.14)
Triglyceride (mmol/L)	1.61 (1.14)
HDL-cholesterol (mmol/L)	1.26 (0.306)
LDL-cholesterol (mmol/L)	2.93 (0.917)
Creatinine (umol/L)	76.3 (19.1)
eGFR (ml/min/1.73 m^2^)	84.0 (11.9)
UACR (ug/mg)	1.43 (1.61)
Sys AOBP (mmHg)	128 (14.8)
Dia AOBP (mmHg)	77.3 (12.1)
ABPM 24 h-SBP (mmHg)	133 (15.0)
ABPM 24 h-DBP (mmHg)	77.3 (11.8)
ABPM Day-SBP (mmHg)	135 (15.6)
ABPM Day-DBP (mmHg)	79.4 (12.4)
ABPM Night-SBP (mmHg)	121 (16.8)
ABPM Night-DBP (mmHg)	68.7 (10.8)

Data are shown as the mean and standard deviation for the continuous variables and frequencies and percentages for the categorical variables. AOBP: Automated office blood pressure, ABPM: Ambulatory blood pressure monitoring.

**Table 2:** Summary of the platelet derived extracellular vesicles, microvascular, and macrovascular damage comparisons between the normotensive and hypertensive patients.

	Hypertensive	Normotensive	*p*
	(*n* = 52)	(*n* = 10)	
Whole superficial vascular plexus density	46.0 (3.01)	46.9 (2.62)	0.37
Fovea superficial vascular plexus density	21.0 (4.98)	18.9 (6.39)	0.35
Parafovea superficial vascular plexus density	48.8 (3.31)	50.0 (2.61)	0.23
Whole deep vascular plexus density	51.3 (3.45)	52.5 (1.93)	0.15
Fovea deep vascular plexus density	36.4 (5.93)	34.4 (7.45)	0.43
Parafovea deep vascular plexus density	52.9 (3.52)	54.2 (2.25)	0.14
Retinal thickness	318 (17.4)	316 (22.0)	0.16
UACR (ug/mg)	1.43 (1.62)	1.43 (1.69)	0.99
PWV (m/s)	8.36 (1.52)	5.77 (1.35)	<0.001
Mean arterial pressure (mmHg)	96.6 (10.8)	84.0 (10.4)	<0.01
Aortic augmentation pressure (mmHg)	9.59 (5.20)	6.50 (8.55)	0.30
Augmentation index (%)	19.3 (12.1)	6.60 (17.5)	0.05
pEV Concentration (pEV/uL)	10.4 (6.70)	6.34 (4.43)	0.03

Data are shown as the mean and standard deviation for continuous variables. UACR: Urinary albumin–creatinine ratio.

**Table 3:** Summary of the platelet derived extracellular vesicles and blood pressure measurements in patients with and without endothelial dysfunction.

		FMD			EndoPAT *	
	Endothelial Dysfunction (*n* = 23)	No Endothelial Dysfunction (*n* = 16)	*p*-Value	Endothelial Dysfunction (*n* = 9)	No Endothelial Dysfunction (*n* = 29)	*p*-Value
Sys AOBP (mmHg)	130 (14.0)	132 (15.3)	0.71	123 (10.9)	133 (14.8)	0.07
Dia AOBP (mmHg)	78.3 (9.88)	77.4 (12.5)	0.80	80.9 (7.74)	76.5 (11.4)	0.29
ABPM 24 h-SBP (mmHg)	134 (10.6)	133 (16.0)	0.90	135 (18.9)	133 (11.0)	0.71
ABPM 24 h-DBP (mmHg)	78.0 (9.80)	73.1 (10.3)	0.14	76.6 (8.26)	75.1 (10.3)	0.70
ABPM Day-SBP (mmHg)	136 (9.80)	133 (17.4)	0.53	133 (20.0)	136 (11.0)	0.57
ABPM Day-DBP (mmHg)	80.0 (10.2)	75.4 (11.1)	0.19	78.4 (9.22)	77.2 (10.7)	0.76
ABPM Night-SBP (mmHg)	123 (18.7)	117 (11.4)	0.28	118 (12.0)	120 (17.3)	0.69
ABPM Night-DBP (mmHg)	68.3 (10.4)	62.4 (9.97)	0.09	65.8 (8.88)	65.3 (10.8)	0.92
pEV Concentration (pEV/uL)	8.51 (5.52)	11.2 (6.74)	0.17	11.1 (8.85)	9.32 (5.17)	0.45

Data are shown as the mean and standard deviation for continuous variables. * EndoPAT data were missing in one patient due to low quality for assessment.

**Table 4:** Summary of the platelet derived extracellular vesicles and blood pressure measurements in patients with and categories of anxiety.

	Low	Moderate	High	*p*-Value
Anxiety category (Trait)	(*n* = 32)	(*n* = 9)	(*n* = 8)	
Sys AOBP (mmHg)	132 (15.8)	136 (15.4)	123 (13.4)	0.17
Dia AOBP (mmHg)	77.3 (12.1)	81.2 (16.1)	79.9 (8.81)	0.66
ABPM 24 h-SBP (mmHg)	136 (12.2)	132 (15.3)	130 (12.4)	0.36
ABPM 24 h-DBP (mmHg)	76.8 (10.7)	77.1 (11.3)	80.1 (9.89)	0.73
ABPM Day-SBP (mmHg)	138 (13.3)	136 (17.5)	134 (11.5)	0.71
ABPM Day-DBP (mmHg)	79.0 (11.4)	79.6 (13.0)	83.3 (9.88)	0.65
ABPM Night-SBP (mmHg)	124 (16.6)	116 (10.4)	109 (11.0)	0.04
ABPM Night-DBP (mmHg)	67.2 (11.0)	66.1 (8.15)	64.4 (9.14)	0.80
Cortisol	282 (96.7)	354 (85.6)	228 (46.7)	0.08
hsCRP	2.97 (4.55)	2.95 (3.11)	5.97 (12.8)	0.52
pEV Concentration (pEV/uL)	9.70 (6.59)	8.62 (3.61)	9.46 (4.80)	0.89
Anxiety category (State)	(*n* = 37)	(*n* = 6)	(*n* = 6)	
Sys AOBP (mmHg)	132 (16.3)	133 (16.1)	128 (12.4)	0.83
Dia AOBP (mmHg)	78.8 (12.4)	74.3 (14.3)	80.0 (11.2)	0.68
ABPM 24 h-SBP (mmHg)	135 (13.3)	132 (7.76)	136 (15.4)	0.85
ABPM 24 h-DBP (mmHg)	77.6 (10.2)	70.8 (12.4)	82.8 (9.02)	0.14
ABPM Day-SBP (mmHg)	137 (14.6)	137 (9.70)	138 (13.1)	0.96
ABPM Day-DBP (mmHg)	79.8 (11.2)	74.3 (14.6)	85.3 (7.74)	0.25
ABPM Night-SBP (mmHg)	122 (15.1)	111 (6.06)	122 (26.1)	0.33
ABPM Night-DBP (mmHg)	67.6 (9.49)	57.5 (7.77)	70.0 (13.2)	0.05
Cortisol	288 (90.7)	295 (118)	220 (65.6)	0.48
hsCRP	2.96 (4.51)	7.47 (13.5)	1.76 (0.766)	0.22
EV Concentration (cEV/uL)	9.08 (6.18)	8.04 (3.58)	13.3 (3.94)	0.21

Data are shown as mean and standard deviation for continuous variables.

## Data Availability

Data cannot be shared.

## Supplementary Materials

The supporting information can be downloaded at: https://www.mdpi.com/article/10.3390/ijms232315150/s1.
