# Supplementary material for: Evaluation of Circulating Platelet Extracellular Vesicles and Hypertension Mediated Organ Damage
Source: Int J Mol Sci. 2022 Dec 2;23(23):15150. doi: 10.3390/ijms232315150 (PMC9741224; doi:10.3390/ijms232315150)
Supplement: Supplementary file 1 [file ijms-23-15150-s001.zip › ijms-2000440-supplementary.pdf]

Supplementary material

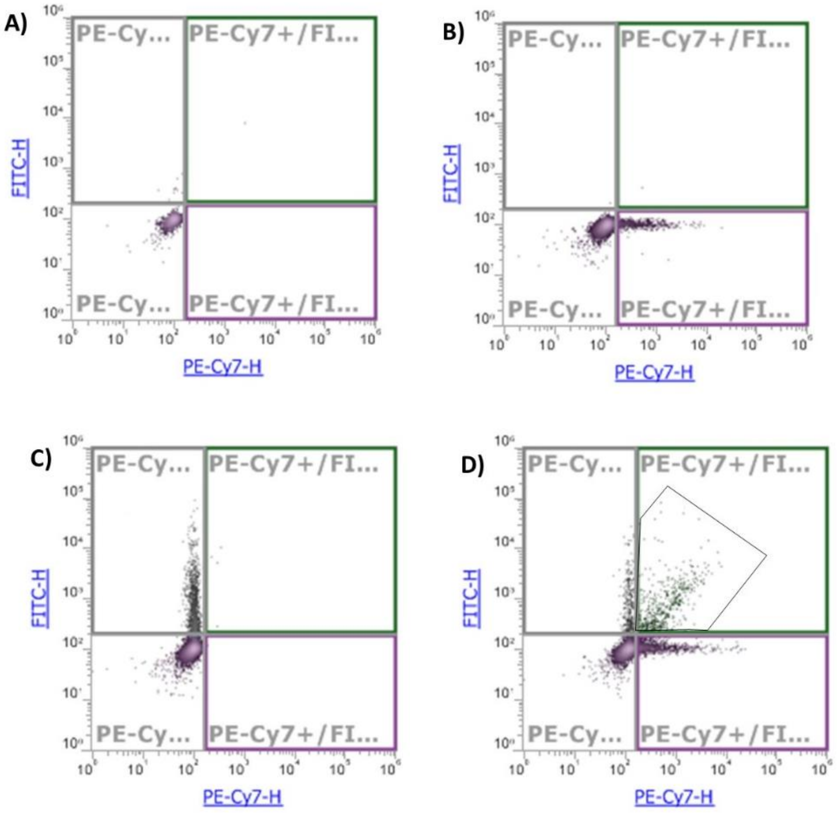

Figure S1. Gating and acquisition strategy for the detection of circulating extracellular vesicles with flow cytometry. A) Buffer only. **B)** CD41-PE Cy7 single stain. **C)** Annexin V-FITC single stain. **D)** Double staining with CD41-PE Cy7 and Annexin V-FITC.
